# Technical and clinical analysis of microEEG: a miniature wireless EEG device designed to record high-quality EEG in the emergency department

**DOI:** 10.1186/1865-1380-5-35

**Published:** 2012-09-24

**Authors:** Ahmet Omurtag, Samah G Abdel Baki, Geetha Chari, Roger Q Cracco, Shahriar Zehtabchi, André A Fenton, Arthur C Grant

**Affiliations:** 1 Bio-Signal Group, 760 Parkside Avenue, Ste 206, Brooklyn, NY, 11226, USA; 2 Departments of Neurology and Pediatrics, State University of New York, Downstate Medical Center, Brooklyn, USA; 3 Department of Neurology, State University of New York, Downstate Medical Center, Brooklyn, USA; 4 Department of Emergency Medicine, State University of New York, Downstate Medical Center, Brooklyn, USA; 5 Center for Neural Science, New York University, New York and Department Physiology & Pharmacology, State University of New York, Downstate Medical Center, Brooklyn, USA; 6 Departments of Neurology, and Physiology & Pharmacology, State University of New York, Downstate Medical Center, Brooklyn, USA

**Keywords:** Electroencephalography (EEG), EEG technology, EEG machine, Signal analysis, Emergency department

## Abstract

**Background:**

We describe and characterize the performance of *microEEG* compared to that of a commercially available and widely used clinical EEG machine. microEEG is a portable, battery-operated, wireless EEG device, developed by Bio-Signal Group to overcome the obstacles to routine use of EEG in emergency departments (EDs).

**Methods:**

The microEEG was used to obtain EEGs from healthy volunteers in the EEG laboratory and ED. The standard system was used to obtain EEGs from healthy volunteers in the EEG laboratory, and studies recorded from patients in the ED or ICU were also used for comparison. In one experiment, a signal splitter was used to record simultaneous microEEG and standard EEG from the same electrodes.

**Results:**

EEG signal analysis techniques indicated good agreement between microEEG and the standard system in 66 EEGs recorded in the EEG laboratory and the ED. In the simultaneous recording the microEEG and standard system signals differed only in a smaller amount of 60 Hz noise in the microEEG signal. In a blinded review by a board-certified clinical neurophysiologist, differences in technical quality or interpretability were insignificant between standard recordings in the EEG laboratory and microEEG recordings from standard or electrode cap electrodes in the ED or EEG laboratory. The microEEG data recording characteristics such as analog-to-digital conversion resolution (16 bits), input impedance (>100MΩ), and common-mode rejection ratio (85 dB) are similar to those of commercially available systems, although the microEEG is many times smaller (88 g and 9.4 × 4.4 × 3.8 cm).

**Conclusions:**

Our results suggest that the technical qualities of microEEG are non-inferior to a standard commercially available EEG recording device. EEG in the ED is an unmet medical need due to space and time constraints, high levels of ambient electrical noise, and the cost of 24/7 EEG technologist availability. This study suggests that using microEEG with an electrode cap that can be applied easily and quickly can surmount these obstacles without compromising technical quality.

## Background

Obtaining rapid EEGs in the ED could improve patient care by narrowing the differential diagnosis and avoiding unnecessary tests, procedures, admissions, and costs. Approximately two to ten percent of all patients presenting to US emergency departments (EDs) present with altered mental status (AMS), with the most frequent underlying cause being neurological disease [1]. Studies show that ED patients with AMS whose initial evaluation includes EEG are diagnosed more accurately and sooner than those without an EEG [2-9]. Despite its utility, routine use of EEG in the ED faces numerous obstacles. Hospital EEG laboratories are rarely open around the clock [10,11]. An informal Internet-based survey found only 2% of EDs are equipped with EEG machines or have a technologist who can properly apply EEG electrodes, troubleshoot problems, and record a technically adequate study. Attaching a full set of EEG electrodes can take up to 30 min and even longer with an uncooperative or agitated patient. The long wires leading from the electrodes to the traditional EEG machine act as antennas and often pick up relatively high-voltage ambient electrical noise because of the large number of noise sources in the ED environment. The electrode wires may also constrain movement and limit access of medical personnel to the patient in the typically cramped emergency department setting. Other reasons for the infrequent use of EEG in the ED include lack of space, cost of EEG machines, and the difficulty of finding skilled EEG interpreters available 24/7 [12].

**Figure 1:**
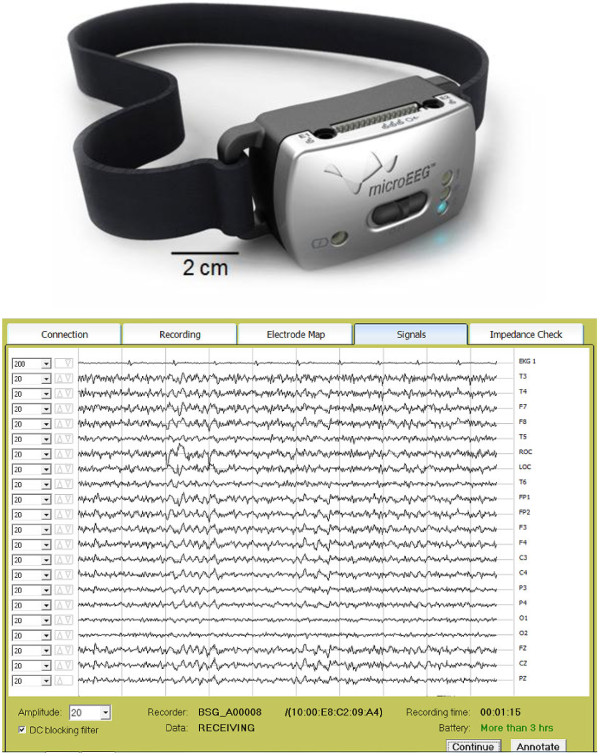
**The microEEG system. **The recorder and transmitter (top) and the interface of the software running on a PC that allows the user to control the microEEG, view the signals, adjust the display scale and filters, check the battery, and enter annotations (bottom).

This article describes a new EEG device (“microEEG”) that can potentially overcome these limitations (Figure 1). microEEG is a miniature, portable, battery-powered, and wireless EEG device. Although each of these qualities is not in itself unique, their combination in a single device that can record high-quality EEG signals from high and unbalanced impedances sets microEEG apart from other available wireless EEG machines. microEEG was developed by Bio-Signal Group, optimized for obtaining high-quality EEG recordings in the ED, and has been certified to meet electromagnetic compatibility (EMC) and medical safety standards. Since the initial review of this article, the microEEG device has received FDA 510(k) approval. The aims of this study were to evaluate both the feasibility of the microEEG for routine use in EDs and the quality of its signals relative to those acquired by a standard, commercially available EEG machine.

## Methods

The microEEG, at merely 9.4 × 4.4 × 3.8 cm and 88 g, is about the size and weight of a cellular telephone and can work equally well with both standard cup electrodes and readily available and rapidly deployable electrode caps, whose electrode-scalp impedances are relatively high. The microEEG digitizes the EEG signals close to the electrodes, transmits the digital data wirelessly to a personal computer located within 10 m, and stores the data on an on-board memory card. Custom software running on the PC controls the device, measures electrode impedances (including ground and reference electrodes), displays the signals and impedances, allows the entry of annotations, and writes data to the hard disk. The software can also stream data to a remote server where an authorized user can review and interpret the EEG.

Table 1 shows the manufacturer’s specifications for the microEEG, Nicolet Monitor (which we use as the comparison or “standard” system), and Trackit, another portable system that we identified as the commercially available device with the closest technical specifications to the microEEG.

**Table 1:** Properties of selected EEG devices

**Product**	**microEEG**	**Nicolet Monitor**	**Trackit Recorder**
Company	Bio-Signal Group	CareFusion Corp.	Lifelines Ltd.
A/D Converter resolution (bits)	16	16	16
Voltage resolution (μV)	0.15	0.15	0.15
Maximum input range	10 mV p.to.p.	10 mV p.to.p.	10 mV p.to.p.
Sampling rate	1,000 Hz	Up to 2,000 Hz	Up to 256 Hz
Bandwidth	0.15 − 500 Hz	0.16 − 1,000 Hz	0.16 − 128 Hz
Input impedance (MΩ)	> 100	> 100	> 100
Separately test GND/REF impedance	Yes	No	No
Number of channels	32	64	12-36 (depending on model)

Four experiments were performed to compare microEEG recordings to those of the standard system, with the EEGs obtained in various clinical environments. Comparisons were made of both technical data and clinical interpretations rendered by a blinded reviewer. The four experiments and the techniques used in the technical comparisons are described in the subsequent five subsections. See the Appendix for additional technical details. The studies were approved by the SUNY Downstate Medical Center IRB, and written informed consent was obtained from all volunteer subjects.

### Parallel recording

A parallel recording was made simultaneously with the microEEG and the standard system, with gold-plated cup electrodes on a healthy adult volunteer subject in the EEG laboratory. This permitted direct comparison of signals between the devices without any possibility of confounding from differences in signal origin. A signal splitter bifurcated the signal from each cup electrode into two identical streams. Prior to the use of the splitter in the EEG recordings, its accuracy was verified with standard engineering techniques, which included delivering currents of varying frequency and amplitude to the electrode leads through a signal generator and comparing the resulting signal in the two streams. Study duration was 15 min.

### microEEG in the EEG Laboratory

Fifteen 20–30-min microEEG recordings were obtained on adult volunteers. Signal properties of these recordings were compared to those of 28 standard EEGs. Time and frequency domain properties of the microEEG data were compared to those of the standard system with all EEGs recorded in the EEG laboratory. The purpose of this experiment was to test the microEEG as a signal acquisition system without changing electrodes or the environment of a typical EEG examination. All EEGs were recorded using standard 9-mm gold-plated cup electrodes placed according to the international 10–20 system. The subject's scalp was rubbed with NuPrep EEG skin prepping gel, and the electrodes were attached using Ten20 conductive paste.

### microEEG in the ED

In the next experiment, 51 microEEG recordings obtained in the ED were compared to standard recordings obtained in the ED and ICU. microEEG recordings were made with the rapidly deployable and commercially available Electro-Cap (23 studies) and with standard gold-plated cup electrodes (28 studies). The Electro-Cap is made of an elastic, spandex-type fabric with recessed, pure tin electrodes attached to the fabric. A small amount of electro-gel was injected through a hole in the center of each electrode to minimize the electrode-scalp impedance. For a realistic clinical simulation of the ED environment, volunteers were connected to noninvasive devices including a pulse oximeter, EKG machine, and oxygen nasal mask. A saline drip was connected to the subject by taping a blunt needle to his or her arm. Blood pressure (BP) monitoring was done by an automated pressure cuff placed around the subject's arm for BP detection every 10 min during the EEG recording. Electrode impedances were measured at the beginning and end of each recording. Standard recordings consisted of 7 ED studies and 11 ICU studies randomly selected from all of the ED and ICU studies obtained in the prior 1 year at SUNY Downstate Medical Center.

### Assessment by a clinical neurophysiologist

A board-certified clinical neurophysiologist reviewed 37 de-identified 30-min EEGs obtained with either the microEEG or the standard system. The data set consisted of 14 microEEG studies recorded in the ED with the Electro-Cap, 13 microEEG studies recorded in the ED with cup electrodes, 8 standard EEGs recorded in the EEG laboratory, and 2 standard EEGs recorded in the ICU. All recordings were reviewed using Insight II, Persyst Development Corp. (Prescott, AZ). The reviewer determined whether each recording was technically adequate for clinical interpretation, i.e., was not substantially obscured by artifacts that rendered the study uninterpretable for clinical purposes.

### Measures of agreement

The simultaneous parallel recording with the microEEG and the standard system provided a unique opportunity to compare the recorded signals in the time domain. We computed the short time correlations between the two signals and examined their values throughout the recording on all channels. The correlations were also used as a guide to focus visual inspection on specific segments of the recording. Standard deviations, higher order statistics, and Hjorth mobility and complexity parameters [13-15] for each system’s signals were also examined.

Frequency domain measures were used to compare microEEG and standard system recordings in all of the experiments. We began by computing the power spectral density (PSD) of each channel over a 500-s interval. Spectral properties were derived from the PSD data.

Combinations of such indicators have been used with varying levels of success to detect both normal and abnormal EEG findings, as well as artifacts. Examples include detection of rhythmic discharges in newborns [16], multi-morphologic ictal patterns in the human long-term EEG [17], muscle and electrical noise artifacts [18], seizure prediction (reviewed by [19]), early patient-specific seizure detection [20], classification of sleep stages [21,22], and identification of resting state [23] or epileptic [24] brain networks.

When sampling rates for the microEEG and standard system were unequal, the microEEG signal was resampled to the time points of the standard system using cubic spline interpolation [25]. Whenever needed (as indicated in the Appendix), the data were bandpass filtered with zero phase shift by a sixth order digital Butterworth filter. This diverse set of measures from both the time and frequency domains provided a comprehensive measure of technical performance.

## Results

**Figure 2:**
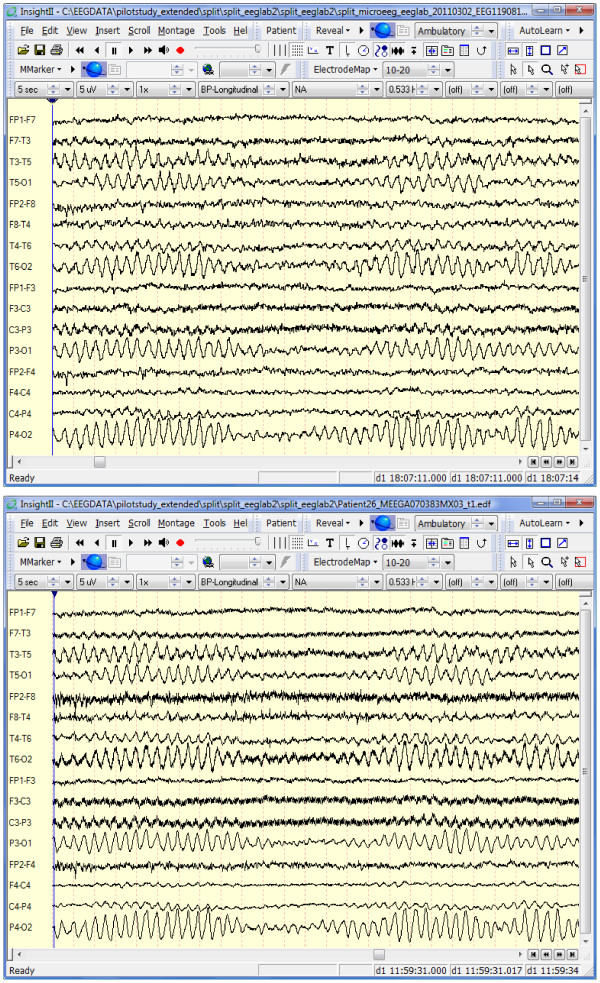
**Typical segment from the EEG signals recorded by microEEG (top) in parallel with the standard system (bottom) shown in a standard EEG viewing environment (Insight II from Persyst Development Corp.). **Insight's high-pass filter is on, and the resolution is set to high. Note that time and amplitude scales as shown in the viewer controls above the EEG traces are, respectively, 5 s and 5 μV.

Visual inspection of the parallel recordings with an AP bipolar longitudinal montage did not reveal any clinically significant difference between the studies. Figure 2 shows a section of the parallel recordings displayed with Insight II (Persyst Corp.). Low-pass and notch filters are off, and the resolution is set to high. The microEEG and standard EEG appear nearly identical, although there is greater high frequency noise in the standard EEG (Figure 2, bottom panel). Visual inspection of the remaining microEEG studies revealed that none of the signals contained unexplained artifacts or expected artifacts (e.g., due to muscle, movement, EKG) at levels greater than those found in the standard recordings.

### Parallel recording

**Figure 3:**
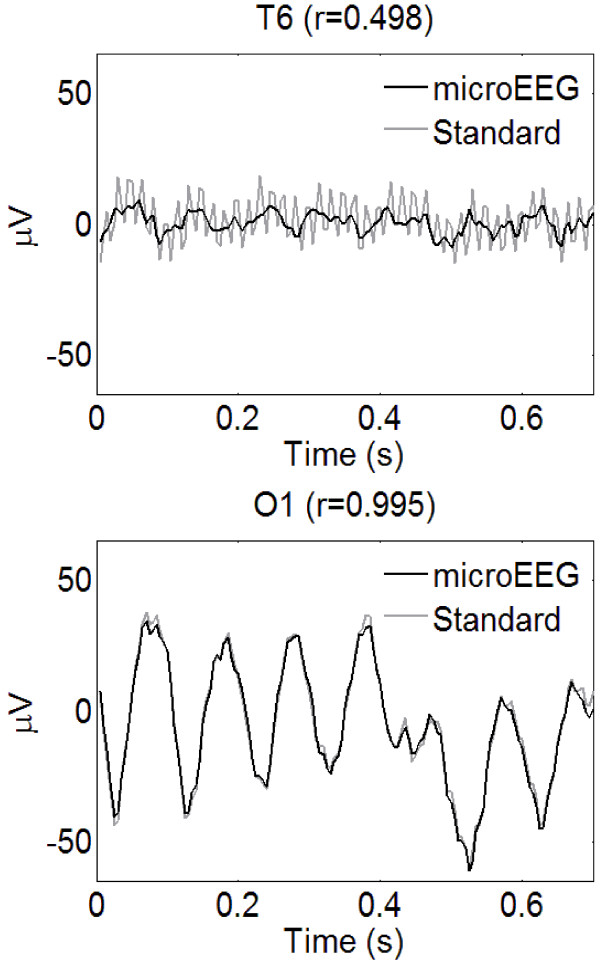
**Superimposed segments of microEEG and standard EEGs recorded in parallel from the same electrodes with a signal splitter. **Electrode and correlation coefficient between the signals during each segment is shown above the plots. The segment from T6 illustrates significantly lower 60-Hz line noise in the microEEG compared to the standard EEG. Filters: 0.50-70 Hz bandpass.

Figure 3 illustrates the superimposition of 0.7 s of simultaneous signal recorded from the microEEG and the standard system at electrodes T6 and O1. These examples were selected to show segments with low (*r* ≈ 0.5) and high (*r* ≈ 1) correlation. The T6 segment illustrates suppression of 60-Hz noise by microEEG. Close examination of all 20 channels throughout the recordings revealed that imperfect correlation was due primarily to relatively greater 60-Hz noise in the standard system.

Table 2 summarizes the statistics of correlation for the entire recording over all electrodes. As expected, correlation was proportional to the amplitude of EEG signals and inversely proportional to 60-Hz noise. For example, the average correlation for channel O2 during an eyes-closed segment of the EEG (when there is relatively high posterior alpha activity) was 0.960 ± 0.047 (mean ±standard deviation).

**Table 2:** Averaged short time correlations

**Notch filter**	**Overall**	**Eyes open**	**Eyes closed**
Off	0.860 ± 0.106	0.849 ± 0.109	0.877 ± 0.099
On	0.911 ± 0.070	0.902 ± 0.076	0.926 ± 0.058

Correlations between themicroEEG and standard EEG computed for 1-s windows and averaged over the recording for all electrodes. Filters: 0.5-70 Hz bandpass.

Table 3 illustrates the sensitivity of time domain parameters to high-frequency noise. Again, the data were obtained from analyzing the signal on all electrodes throughout the recording. In particular, Hjorth mobility was higher in the standard EEG than microEEG and highly sensitive to high frequency noise. This sensitivity is expected since it is interpretable as the standard deviation of the PSD [14]. By contrast, higher order statistics and Hjorth complexity were unchanged when 60-Hz noise was removed from the signals. The skewness was not significantly different from zero and is not shown. Note that we have defined kurtosis as the fourth central moment of the signal divided by the square of the variance so that kurtosis equals 3 for a normal distribution.

**Table 3:** Time and frequency domain properties

	**Time domain**				**Spectral**		
	**Stdev(μV)**	**Kurt**	**Mob (Hz)**	**Comp**	**MF (Hz)**	**SEF75 (Hz)**	**SE**
mEEG (Notch off)	67.9	9.1	108.5	2.9	9.3	9.4	11.8
STD (Notch off)	74.7	6.9	149.4	2.3	15.6	29.0	11.4
mEEG (Notch on)	65.6	10.0	69.0	3.5	6.0	7.9	11.9
STD (Notch on)	67.9	8.9	71.8	3.2	6.8	9.4	12.0

microEEG and standard EEG recorded in parallel. Filters 0.5-70-Hz bandpass. Data shown are averages over all channels. mEEG, microEEG; STD, standard; Stdev, standard deviation; Kurt, kurtosis; Mob, mobility; Comp, complexity; MF, mean frequency; SE, spectral entropy. Kurtosis, mobility, complexity, and spectral entropy are dimensionless.

**Figure 4:**
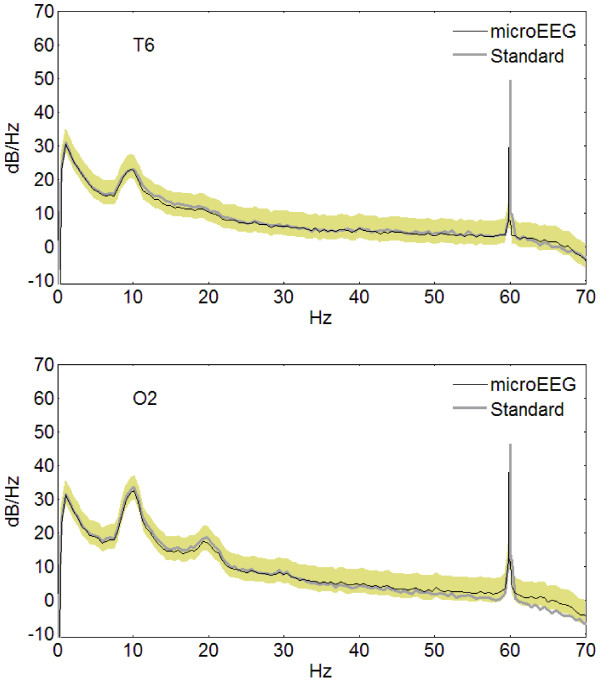
**Examples of the power spectral density of microEEG and standard EEG signals recorded in parallel. **The curves were smoothed except near 60 Hz. The shaded zone represents the 95% confidence range for the microEEG signal (the 95% confidence range for the standard EEG was essentially the same and is not shown). Filters: 0.50-70-Hz bandpass.

Figure 4 shows the PSD of the signal recorded by the microEEG and standard system on two channels, T6 and O2. These channels contained a significant difference in 10-Hz activity and were selected to illustrate this difference. The curves are smoothed for better visibility except in the neighborhood of 60 Hz. Inspection of the PSD for all channels showed that the standard signal’s spectrum agreed well with the microEEG spectrum and was within the confidence limits in all channels and frequencies. Quantities derived from the PSD also had a range of sensitivities to high frequency noise as demonstrated by the results in Table 3. Note that notch filtering both signals drastically reduced the difference in SEF75 and resulted in good agreement.

### microEEG in the EEG Laboratory

Comparison of the band power of the microEEG and standard system indicated that there was good agreement in all frequency ranges except in the lowest range, 0–4 Hz, and at 60 Hz. The mean band power of each channel averaged across recordings was also examined and found to have a similar agreement between the two devices. In the ranges alpha–beta4, the two systems’ band powers were nearly equal, while in the theta and beta5 ranges they were within one standard deviation of each other. The standard deviations were calculated from the variability across recordings and channels. Lower power in the 0–4 Hz band in the microEEG recordings was due to the difference in the hardware high-pass filters: the low frequency cutoff in the microEEG was set at 1 Hz in these experiments compared to 0.16 Hz in the standard device. Lower 60-Hz noise in the microEEG compared to the standard system likely resulted from the shorter EEG electrode cable lengths as the common mode rejection ratio of the two systems is the same. This experiment demonstrated that microEEG signals do not contain activity at levels that are unexpectedly different in any frequency range from those recorded by the standard device.

### microEEG in the ED

**Figure 5:**
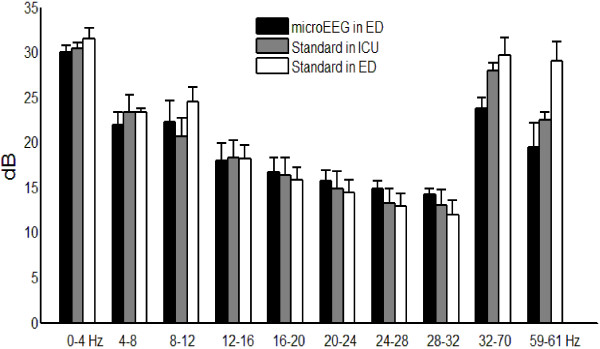
**Band power for the microEEG and standard system. **microEEG with Electro-Cap in the ED (28 recordings), standard system in the ICU (11 recordings), and standard system in the ED (7 recordings). Error bars indicate the standard deviation of variability due to differences among channels and recordings. Filters 0.5-70 Hz bandpass.

Figure 5 shows that there was good agreement between the microEEG and the standard system in all frequency bands except at 60 Hz, where the microEEG power was less. The agreement at 0–4 Hz is much better than in the recordings from the EEG laboratory because the microEEG hardware high-pass cutoff was set to 0.15 Hz. The microEEG recordings with the cup electrodes had results (not shown) similar to those with the Electro-Cap with the exception of somewhat higher power near 60 Hz. The mean impedance of Electro-Cap electrodes was 15.9 ± 17.6 kΩ with median 8 kΩ and range 0.9 to 80 kΩ. The mean impedance of cup electrodes was 5.3 ± 4.2 kΩ with median 4.3 kΩ and range 1.2 to 33.3 kΩ. The impedance of each channel is taken as the mean of the impedance at the start and end of each recording. The inter-recording variability of impedance was greater than the intra-recording variability. For each recording, the value of the impedance at the end of a recording was strongly correlated to the value at the start *r* = 0.84 (Electro-Cap) and 0.83 (cup electrodes)]. We note that good agreement between the microEEG in the ED and the standard EEG was achieved even though the electrode cap electrode impedances were generally substantially greater than the < 5 kΩ impedance IFCN standard for digital EEG machines [26].

### Assessment by a clinical neurophysiologist

Two board-certified clinical neurophysiologists independently evaluated the technical quality of ten randomly selected microEEG recordings done in the ED and described in this article (equally divided between cup electrodes and Electro-Cap); they found all of them suitable for making clinically significant interpretations. We then designed the blinded study described in Methods. The blinded reviewer was asked, “Is the recording clinically acceptable?" The results show that the microEEG in the ED performed better than the standard system in the ICU but not as well as the standard system in the EEG laboratory (Table 4). The percentage of Yes answers for the microEEG was significantly lower than for the standard EEG from the EEG laboratory (z = 4.6, *p* < 0.05 test of proportions) but not different from the standard EEG if the EEG laboratory and ICU recordings were not distinguished (z = 1.21, *p* > 0.05 test of proportions). Reasons given for clinically unacceptable recordings included wavering baseline, sharply contoured artifacts especially in T5 and T6, excessive EKG artifacts, 60-Hz artifacts, bursts of diffuse artifacts, and muscle artifacts on all leads.

**Table 4:** Responses given to the question "Is the recording clinically acceptable?"

	**Yes (%)**	**Maybe (%)**	**No (%)**
microEEG/E-Cap/ED	60	27	13
microEEG/Cup/ED	58	34	8
Standard/EEG lab	88	12	0
Standard/ICU	0	50	50

Blinded evaluation by a neurologist of a set of recordings that contained both microEEG and standard recordings.

## Discussion

Over the past 2 decades, EEG technology has improved dramatically. Large analog machines with paper recordings have been replaced by much smaller, computer-based digital machines, with all the associated advantages of digital recording and data storage. Preamplifier input impedances have risen without sacrificing CMRR. Despite these advances, recording a technically acceptable EEG in electrically hostile environments such as the ED remains a challenge, especially with uncooperative patients in the cramped confines of a busy ED. The most significant challenges are the following: (1) high line noise (60 Hz) in the recorded signals due to high ambient noise levels, long electrode wires, and relatively high electrode impedances and inter-electrode impedance differences; (2) time needed to attach a full set of EEG electrodes and achieve low electrode-scalp impedances; (3) around the clock availability of trained EEG technologists; and (4) limiting physical access to the patient with the electrode wires and EEG equipment.

This study demonstrates that the microEEG can overcome all of these obstacles. Its miniature size, a built-in rechargeable battery power source, and wireless transmission of digitized EEG data eliminate the physical access problems and space requirements associated with EEG electrode wires, recording equipment, and power cables. The engineering specifications of the microEEG (e.g., A/D converter resolution, sampling rate, input impedance, CMRR, number of channels, etc.) are comparable to currently available commercial systems. However, because the microEEG is wireless and small enough to be rigidly attached to the patient, for example on the head using a headband or on an electrode cap, it can be implemented with very short electrode wires. The short cables, combined with the on-board DC power source, resulted in microEEG signals having less contamination with 60-Hz noise than did those of the standard system. The lower line noise in microEEG signals was apparent in the simultaneous parallel recording from a volunteer subject with standard cup electrodes (Figures 2, 3, 4). It was also seen across 28 recordings obtained in the ED with the microEEG and the Electro-Cap compared to 18 recordings made with the standard system and cup electrodes in the ED or ICU (Figure 5).

These experiments also demonstrated high concordance between the microEEG and the standard system of spectral properties in the frequency range of physiologic EEG activity (Figures 4 and5 and Table 3, “spectral” columns with notch filter on). Not surprisingly, there was also high agreement in the time domain between the microEEG and standard system recordings when the difference in 60-Hz noise was reduced with the notch filter (Table 2 and Table 3, “time-domain” columns, notch filter on). The fact that notch filtering the signals caused the mean levels of all measures to become nearly equal between the two systems (Table 3), combined with the data shown in Figure 3, demonstrates that the source of differences between the systems was the relatively higher line noise in the standard system. In other words, comprehensive frequency and time domain analyses of EEG signals recorded with the microEEG and standard system did not reveal any device-specific differences other than the generally higher 60 Hz noise in the standard system. Thus, advantages deriving from the microEEG’s ease of use can be obtained without compromise by substituting the microEEG for a standard EEG machine when ≤ 26 recording channels are needed.

The absence of systematic differences in signal properties between the microEEG and standard system is reflected in the blinded assessment of EEGs from both systems by an experienced clinical neurophysiologist. As shown in Table 4, the fraction of microEEG studies recorded in the ED with either cup electrodes or the Electro-Cap considered definitely acceptable for clinical interpretation was 58 and 60%, respectively, compared to 88% for EEGs recorded with the standard system and cup electrodes in the EEG laboratory and 0% of standard system studies from the ICU. Perhaps equally important, the fraction of clinically acceptable (as well as possibly acceptable) microEEG studies in the ED did not differ significantly between those recorded with cup electrodes and the Electro-cap. In a separate prospective study of ED patients presenting with altered mental status at our institution, patients receive both a standard EEG with cup electrodes and a microEEG with the Electro-Cap. Data from the microEEG recordings in the prospective study are not yet available for analysis.

These data reveal a significant additional advantage of the microEEG – its ability to generate high-quality EEG from electrodes with high electrode-scalp impedances. Specifically, when used with the Electro-Cap, the microEEG performed well with electrode impedances substantially higher than the 5 kΩ recommended by professional societies, i.e., mean 15.9 ± 17.6 kΩ (range 0.9 to 80 kΩ) [26] and with interelectrode impedance differences within an EEG much higher than the 2 kΩ reported to degrade CMRR, i.e., mean 8.7 ± 11.2 kΩ (range 0 to 60 kΩ) [27]. This result provides a mechanism to overcome the two remaining obstacles listed above to achieving quality EEG recordings in the ED environment. In the prospective study mentioned above, the mean setup time (i.e., time between initial contact with the patient and beginning the EEG recording) was 13 ± 7 min for the first 50 patients, nearly all of whom were uncooperative and many of whom were agitated. There is also no risk of an agitated patient accidentally dislodging or pulling off electrode wires after the electrodes are attached. In a later phase of this study, the microEEG and Electro-Cap hardware will be used by personnel with minimal formal training in EEG electrode placement and EEG equipment (e.g., EKG technicians) to obtain EEGs on ED patients with AMS. However, the data already obtained with EEG technologists in the ED [28], and with non-EEG technologists applying EEG electrodes in other research studies on healthy subjects [29], strongly suggest that the microEEG technology in combination with readily available and rapidly deployable electrode arrays will permit rapid acquisition of EEGs in the ED by personnel without the training and experience of EEG technologists. Since the ED is one of the most challenging environments for recording EEG, we expect that these results will be relevant for recording EEG in other challenging environments such as ICUs, as well as in the EEG laboratory.

A limitation of this study was that it used recordings obtained only from healthy adult volunteers and consequently involved no abnormal EEG patterns. This followed from the fact that it was designed as an initial pilot study to demonstrate the feasibility and safety of the microEEG as a prerequisite to a separate study of its diagnostic accuracy with patients.

## Conclusions

microEEG is a miniature, wireless, battery-powered EEG device with engineering specifications equivalent to those of much larger commercially available EEG machines.

The analog EEG signals are amplified and digitized within the device, and then transmitted wirelessly to a laptop computer within 10 meters. Comprehensive time and frequency domain analyses of microEEG recordings from normal volunteers in an EEG laboratory and emergency department revealed neither unexpected signals nor significant differences from EEGs recorded with a Nicolet Monitor machine. microEEG is typically placed on or near the patient’s head and can record high-quality EEG from electrodes with high electrode-scalp impedances. Its noise immunity is enhanced by the short length of electrode wires from scalp to device. This feature is particularly useful when the device is used with an electrode cap, or in a setting such as the emergency department where time and space constraints may limit a technologist’s ability to achieve and maintain low electrode impedances throughout the recording.

## Appendix A

The following 20 channels were used in the parallel recording: FP1, FP2, F3, F4, C3, C4, P3, P4, O1, O2, F7, F8, T3, T5, T6, FZ, CZ, PZ, LOC, and ROC. Sampling rates for the microEEG and standard system were 200 and 500 Hz, respectively. For the recordings with the microEEG in the EEG laboratory, we used the following 16 channels: FP1, FP2, F3, F4, C3, C4, P3, P4, O1, O2, F7, F8, T3, T4, T5, and T6. The sampling rate was 250 Hz. Signals from electrodes F7, F8, T5, and T6 were used in the analyses for the microEEG in the ED. In computing the power spectral density (PSD), the multitaper method [30] with six orthogonal tapers was used, with the size of the time series defined as the length of the FFT (Matlab’s function *pmtm*). The spectral properties derived from EEG data were as follows: band power: the integral of the PSD over specified frequency ranges. The ranges used were delta (0 ≤ f < 4 Hz), theta (4 ≤ f < 8 Hz), alpha (8 ≤ f < 12 Hz), beta1 (12 ≤ f < 14 Hz), beta2 (14 ≤ f < 16 Hz), beta3 (16 ≤ f < 20 Hz), beta4 (20 ≤ f < 24 Hz), beta5 (24 ≤ f < 32 Hz), high frequency (32 ≤ f < 70 Hz), and power line noise (59 ≤ f < 61 Hz). Band power can be considered as a frequency-specific contribution to the overall variance of the signal. Mean frequency: the first moment of the normalized PSD. SEF75: The 75% spectral edge frequency, that is, the frequency that contains 75% of all the power in the spectrum (starting at f = 0 Hz). Spectral entropy: the entropy of the normalized PSD interpreted as a discrete probability distribution. This ranges between 0 for a maximally concentrated PSD tothe value log_2_(N), for a uniform distribution, where N is the total number of discrete frequencies in the PSD (N was constant for all channels and for both systems).

## Competing interests

Supported by NIH grant 1RC3NS070658 to Bio-Signal Group (BSG), with a subcontract to SUNY Downstate Medical Center. André Fenton is a founder of BSG. Samah Abdel-Baki and Ahmet Omurtag are employees of Bio-Signal Group and with André Fenton are co-inventors listed on a patent application based on the microEEG (pub. no. WO/2010/129026. Title: EEG Kit). ACG serves on the BSG advisory board. All income derived from this position is donated directly from BSG to the Downstate College of Medicine Foundation.

## Authors’ contributions

Drs. Abdel Baki and Omurtag contributed equally to this paper. All authors read and approved the final manuscript.
